# Use and uptake of web-based therapeutic interventions amongst Indigenous populations in Australia, New Zealand, the United States of America and Canada: a scoping review

**DOI:** 10.1186/s13643-020-01374-x

**Published:** 2020-05-31

**Authors:** Rachel Reilly, Jacqueline Stephens, Jasmine Micklem, Catalin Tufanaru, Stephen Harfield, Ike Fisher, Odette Pearson, James Ward

**Affiliations:** 1 Aboriginal Health Equity, South Australian Health and Medical Research Institute, Adelaide, South Australia Australia; 2 College of Medicine and Public Health, Flinders University, Bedford Park, South Australia Australia; 3 School of Medicine, University of Adelaide, Adelaide, Australia; 4 Rosemary Bryant AO Research Centre, Clinical and Health Sciences, University of South Australia, Adelaide, South Australia Australia; 5 Australian Institute of Health Innovation, Macquarie University, Sydney, New South Wales Australia; 6 Institute of Urban Indigenous Health, Windsor, Queensland Australia

**Keywords:** e-mental health, Digital technology, Aboriginal and Torres Strait Islander, Therapeutic intervention, Mobile health, e-health, Mental health, Chronic disease, Infectious disease, Information Technology, App

## Abstract

**Background:**

Barriers to receiving optimal healthcare exist for Indigenous populations globally for a range of reasons. To overcome such barriers and enable greater access to basic and specialist care, developments in information and communication technologies are being applied. The focus of this scoping review is on web-based therapeutic interventions (WBTI) that aim to provide guidance, support and treatment for health problems.

**Objectives:**

This review identifies and describes international scientific evidence on WBTI used by Indigenous peoples in Australia, New Zealand, Canada and USA for managing and treating a broad range of health conditions.

**Eligibility criteria:**

Studies assessing WBTI designed for Indigenous peoples in Australia, Canada, USA and New Zealand, that were published in English, in peer-reviewed literature, from 2006 to 2018 (inclusive), were considered for inclusion in the review. Studies were considered if more than 50% of participants were Indigenous, or if results were reported separately for Indigenous participants.

**Sources of evidence:**

Following a four-step search strategy in consultation with a research librarian, 12 databases were searched with a view to finding both published and unpublished studies.

**Charting methods:**

Data was extracted, synthesised and reported under four main conceptual categories: (1) types of WBTI used, (2) community uptake of WBTI, (3) factors that impact on uptake and (4) conclusions and recommendations for practice.

**Results:**

A total of 31 studies met the inclusion criteria. The WBTI used were interactive websites, screening and assessment tools, management and monitoring tools, gamified avatar-based psychological therapy and decision support tools. Other sources reported the use of mobile apps, multimedia messaging or a mixture of intervention tools. Most sources reported moderate uptake and improved health outcomes for Indigenous people. Suggestions to improve uptake included as follows: tailoring content and presentation formats to be culturally relevant and appropriate, customisable and easy to use.

**Conclusions:**

Culturally appropriate, evidence-based WBTI have the potential to improve health, overcome treatment barriers and reduce inequalities for Indigenous communities. Access to WBTI, alongside appropriate training, allows health care workers to better support their Indigenous clients. Developing WBTI in partnership with Indigenous communities ensures that these interventions are accepted and promoted by the communities.

## Background

Indigenous populations in Australia, New Zealand, the United States of America (USA) and Canada carry a greater burden of ill-health than the general populations in their respective countries [1]. In each of these countries, Indigenous populations also experience barriers to receiving optimal health care due to mistrust of the health system resulting from historic and current mistreatment, language and cultural differences and living in geographically remote locations [1]. Globally, developments in health information and communication technologies (ICT) have been applied to overcome such barriers and to enable greater access to basic and specialist care [2].

In recent years, technological advances have also led to the development of therapeutic interventions delivered electronically for a range of health conditions. The focus of this review is on web-based therapeutic interventions (WBTI), which are self-guided or clinician-assisted programmes delivered via the internet that aim to provide guidance, support and treatment for health problems. The proliferation of mobile devices, including smart phones and electronic tablets, means that web-based programmes and mobile applications (“apps”) can be accessed at low cost by a range of populations, including culturally and linguistically diverse, or other populations who may be otherwise disengaged with the health system for a range of reasons.

In Australia, despite the challenges inherent in remote living, compounded by socioeconomic disadvantage, Aboriginal and Torres Strait Islander populations have high rates of social platform use, indicating high levels of internet connectivity [3]. The recent roll-out of the “National Broadband Network” (NBN) in non-urban areas and other advances in technology have resulted in the rise of telecommunication access in remote areas, where internet access was previously limited [4]. In recent years, the increase in use of social platforms has been significantly greater amongst Aboriginal and Torres Strait Islanders compared with that for all Australians, and a high level of engagement with social platforms has been evident in the health sector [5–7]. Together, advancements and high level use of ICT by Indigenous Australians across all areas of remoteness and sociodemographic spectrum clearly indicate a potential to engage with individuals directly through social platforms, particularly with younger people.

The use of digital health platforms has been advocated to incorporate a wider approach to include social determinants of health and wellbeing [5], given the feasibility of capturing personalised health-related social and behavioural information that was not previously accessible, with direct implications to people living with chronic diseases. Worldwide, there are over 250,000 different consumer-targeted mobile health apps, though few have been rigorously evaluated regarding the accuracy of information they provide, privacy and digital security protections, and their usability, functionality and effectiveness in the context of health [8]. As the use of online therapies continues to grow, this review of international evidence on WBTI for health conditions amongst Indigenous populations is timely.

A previous scoping study has examined the evidence for the effectiveness of web-based and mobile technologies in health promotion, specifically to reach Indigenous Australians [9]. This valuable piece of work focused on social media and mobile apps primarily for smoking cessation, many of which did not appear in the peer-reviewed literature. The current review provides an update on the peer-reviewed evidence of the acceptability, validity and effectiveness of WBTI for a broad range of health conditions, extending the review to include WBTI developed with Indigenous people internationally for communicable and non-communicable diseases, mental health conditions (including the broader concept of social and emotional wellbeing), or issues relating to the use of harmful substances, and problem gambling. The objective of this scoping review is to identify and describe the available international scientific evidence on WBTI used by Indigenous peoples in Australia, New Zealand, Canada and USA for managing and treating health conditions. These four countries were included due to similar persistent patterns of inequities that have arisen in these countries since colonisation, as well as geographic and demographic similarities such as remoteness from health services and differing language, culture and concepts of health and illness from the dominant culture [1]. Additionally, these four countries have other commonalities which allow for comparison; they are developed, democratic, wealthy countries with similar standard of living and life expectancy [10].

## Methods

The study protocol for this scoping review has been published previously [11]. Methods followed the procedures outlined by the Joanna Briggs Institute [12, 13] with reporting adhering to the Preferred Reporting Items for Systematic Reviews and Meta-analyses (PRISMA) guidelines scoping review extension [14]. The relevant PRISMA checklist is included as an additional file.

### Information sources

A systematic search was conducted of the following databases: PubMed, CINAHL, Embase, ATSIHealth via Informit online, Web of Science, APAIS Health databases, Australian Indigenous Health InfoNet and the Primary Health Care Research Information Service (PHCRIS). A search for unpublished studies was conducted by accessing Mednar, Trove, Google, OCLC WorldCatDissertations and Theses, and Proquest Dissertations and Theses. The search in Trove was limited to theses only, as the assumption was made that other publication types would be captured in the other databases. To capture additional literature, a search of websites and clearing houses that provided information, links and resources relating to Indigenous health in each of the four countries that are the focus of this review was conducted using initial keywords including the following: Indigenous, Aboriginal, Torres Strait Islander, Maori, First Nations, First Peoples, Metis, Inuit, Native American, eHealth, telehealth, internet-based intervention and web-based therapeutic tool.

### Search strategy

As outlined in the scoping review protocol [11], a four-step search strategy was followed in consultation with a research librarian, with a view to finding both published and unpublished studies. An initial limited search of PubMed was undertaken. The final search strategy for PubMed can be found in Table 1. An analysis of the text words contained in the titles and abstracts, and of the index terms used to describe articles then informed the development of search strategies tailored for each information source. A second search using all identified keywords and index terms was undertaken across all other information sources. The reference list of all studies selected for inclusion was screened for additional studies. The search strategy allowed for authors to be contacted and experts consulted with a view to accessing any unpublished data or for clarification of published information; however, this was not necessary in practice. The search was conducted on 10 April, 2019.

**Table 1:** Search Strategy for PubMed

Category	Search terms
Population of interest	((“Oceanic Ancestry Group”[mh] OR Aborig*[tw] OR Indigen*[tw] OR (Torres Strait[tw] AND Islander*[tw]) OR “health services, indigenous”[mh]) AND (.au[ad] OR australia[ad] OR Australia[mh] OR Australia[tiab] OR Northern Territory[tiab] OR Northern Territory[ad] OR Tasmania[tiab] OR Tasmania[ad] OR New South Wales[tiab] OR New South Wales[ad] OR Victoria[tiab] *OR* Victoria[ad] OR Queensland[tiab] OR Queensland[ad])) OR Native American[tiab] OR Maori*[tiab] OR (indigenous[tiab] AND new zealand[tiab]) OR “Indians, North American”[mh] OR “Inuits”[mh] OR Inuit*[tiab] OR first nation*[tiab] OR “Alaska Natives”[mh] OR metis[tiab] OR Eskimo*[tiab] OR Canada[mh] OR United States[mh]
Intervention	“telemedicine”[mh] OR telemed*[tiab] OR ehealth[tiab] OR e-health[tiab] OR web*[tiab] OR internet[tiab] OR computer*[tiab] OR mobile[tiab] OR app*[tiab] OR apps[tiab] OR blog*[tiab] OR “social media”[tiab] OR iphone*[tiab] OR smartphone*[tiab] OR “smart phone*”[tiab]
Health issues	(“mental disorders”[mh] OR “gambling”[mh] OR “chronic disease”[mh] OR “exercise”[mh] OR “delivery of health care”[mh] OR “self care”[mh] OR “disease management”[mh] OR “nutrition therapy”[mh] OR “health behavior”[mh] OR “psychotherapy”[mh] OR “patient acceptance of health care”[mh] OR depress*[tiab] OR anxiety[tiab] OR suicid*[tiab] OR “stress management[tiab] OR addict*[tiab] OR drug*[tiab] OR methamphetamine*[tiab] OR alcohol*[tiab] OR ice[tiab] OR meth[tiab] OR amphetamine*[tiab] OR heroin[tiab] OR inhalant*[tiab] OR marijuana[tiab] OR cannabis[tiab] OR morphine[tiab] OR buprenorphine[tiab] OR methadone[tiab] OR speed[tiab] OR crystal[tiab] OR LSD[tiab] OR ecstasy[tiab] OR cocaine[tiab] OR GHB[tiab] OR MDMA[tiab] OR ketamine[tiab] OR solvent*[tiab] OR opioid*[tiab] OR opiate*[tiab] OR narcotic*[tiab] OR illicit[tiab] OR binge drink*[tiab] OR tobacco[tiab] OR smok*[tiab] OR wellbeing[tiab] OR well being[tiab] OR nutrition[tiab] OR diet[tiab] OR medication[tiab] OR adherence[tiab] OR complian*[tiab] OR self manag*[tiab] OR pain manag*[tiab])

### Inclusion criteria

#### Date range

Studies published from 2006 to 2018 (inclusive) in English were considered for inclusion. This timeframe was considered sufficient to capture up-to-date evidence on WBTI that have grown in popularity in recent years.

#### Publication status

Peer-reviewed and grey literature meeting the inclusion criteria were considered for inclusion.

#### Participants

Studies assessing WBTI designed for Indigenous peoples of any age in Australia, Canada, USA and New Zealand were considered for inclusion in the review. Participants could be accessing the WBTI to prevent, manage or treat their own health condition; or could be healthcare providers, friends or family members accessing a WBTI to assist an Indigenous person with a health condition. Studies were considered if more than 50% of participants were Indigenous, or if results were reported separately for the Indigenous participants.

#### Concept

The focus of this review was on the use and uptake of WBTI by Indigenous people. The range of possible health issues the WBTI could address was deliberately broad and included chronic physical illness, communicable disease, mental health conditions and issues relating to social and emotional wellbeing, use of harmful substances or gambling. Studies were considered for inclusion if they provided information on WBTI used by individuals or groups: either autonomously or with assistance, to assess, manage or treat health conditions by (a) modifying lifestyle behaviours, (b) promoting social and emotional wellbeing and resilience, (c) supporting adherence to treatment regimens, (d) increasing motivation to reduce risky behaviours and (e) providing and supporting strategies to reduce dependence on alcohol, prescription drugs, illicit substances or gambling. Evaluations of websites that only provide health education without any interactive or therapeutic content were excluded. This included interventions involving health information kiosks or telehealth as the sole intervention strategy.

#### Context

Health-related WBTI accessed in any setting in Australia, New Zealand, Canada and USA were included.

### Selection of sources

Following the search, all identified citations were uploaded into Endnote^TM^ (Version X8.1, Clarivate Analytics, Philadelphia, USA) and duplicates removed. Citations were then entered into an online systematic review management system (www.covidence.org, 2019, Veritas Health Innovation Ltd, Melbourne, Australia). Titles and abstracts were screened against the inclusion criteria by one researcher (RR), as well as independently by one of two other researchers (SH, IF). Disagreements were resolved through discussion between all three reviewers. Studies that potentially met the inclusion criteria were retrieved in full and imported into Covidence for full text review. The full texts of selected studies were assessed in detail against the inclusion criteria by at least two reviewers (RR, and either SH, FS or IF). Full text studies that did not meet the inclusion criteria were excluded and reasons for exclusion recorded.

### Data charting and synthesis

Two data extraction tools were developed for this scoping review [11], which were further refined during the data charting process. This tool facilitated the extraction of the data mapped to the variables outlined in Table 2. Extracted data was synthesised and reported under four main conceptual categories: (1) types of WBTI used, (2) community uptake of WBTI, (3) factors that impact on uptake and (4) conclusions and recommendations for practice.

**Table 2:** Data extraction variables

Variable name	Description
Author	The surname of the first author of the source publication.
Year	Year study published.
Country	Country and population where the study was conducted.
WBTI	Name of the web-based therapeutic intervention.
Aim	Purpose of the WBTI (e.g. treatment, education, adherence, chronic disease management)
Health condition	The health condition targeted by the WBTI.
Participants	Descriptive demographics about the study population
Delivery mode	WBTI components, frequency intensity and duration of use, self or clinician administered, or clinician assisted
Context	The setting where the study was conducted (e.g. health service or community setting, geographic location (urban, rural, remote), other relevant details reported by authors.
Design	The study design.
Outcomes	The key outcome measures for the study.
Results	Whether the WBTI had a positive or negative change, or no change.
Explanation	Explanations provided by the authors for uptake and treatment effects.
Findings	Author’s conclusions, interpretations and recommendations.

## Results

### Sources of evidence

**Fig. 1:**
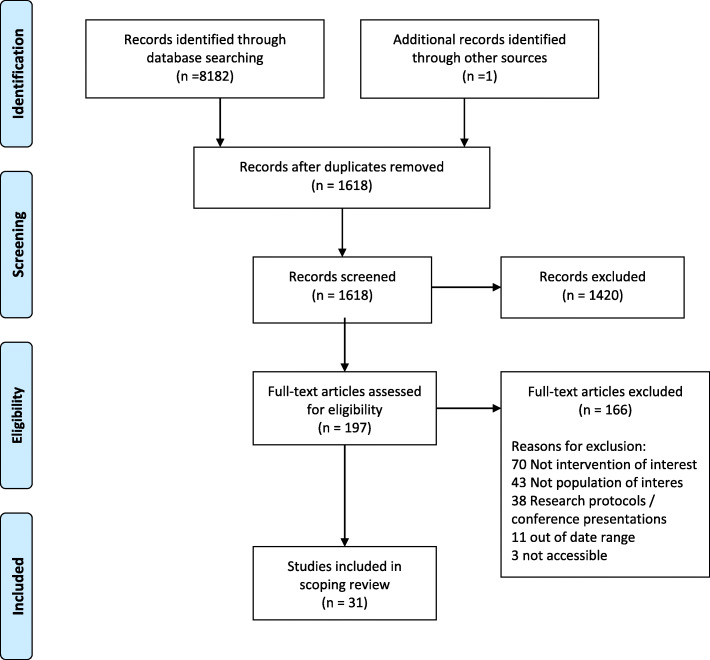
PRISMA flow diagram of the complete search process

The systematic searches identified 8182 references and one additional reference was identified through other sources (Fig. 1). After duplicates were removed, there were 1618 unique papers for review. The large number of duplicates stems from many studies being duplicated across multiple databases. Title and abstract screening excluded 1420 references, and a further 166 papers were excluded on full-text review, resulting in 31 studies meeting the inclusion criteria for this scoping review.

### Characteristics of sources

As shown in Table 3, 11 (34%) sources were from the USA [15–25] and nine (28%) sources were from New Zealand [26–34], with the remainder from Australia (12, 38%) [35–45]. As there were no studies from Canada, the findings reported from here on pertain to New Zealand, the USA and Australia. The majority reported evaluation studies (52%) [16–18, 22, 23, 25, 29, 31, 32, 34–36, 38, 39, 43, 45] and had prospective (94%) data collection [15–28, 30–33, 35–43, 45]. There was an increasing number of publications over time. As shown in Table 4, health topics addressed in the reported studies were varied; however, mental health (32%) [24, 27, 31, 32, 36, 37, 39, 41–43] and substance use (19%) [16, 18, 21, 22, 28] were the two prominent issues targeted. Most sources reported studies that focused on interventions with consumers (82%), although four (18%) [24, 34, 36, 37, 39, 43] sources reported the use of WBTI to support healthcare workers. There were no clear patterns of similarity or differences between countries in any of the variables reported.

**Table 3:** General characteristics of included publications

Characteristic	Number	Percentage (%)
**Country**		
New Zealand	9	29 %
Australia	11	36 %
USA	11	36 %
**Publication year**		
2006–2009	4	13%
2010–2014	10	32%
2015–2018	17	55%
**Study type**		
Case study	1	3%
Evaluation study	16	52%
Feasibility trial	4	13%
Observational study	3	10%
Qualitative study	2	6%
Randomised trial	5	16%
**Study design**		
Prospective	29	94%
Retrospective	2	6%
**Topic**		
Cardiac care	3	10%
Diabetes	3	10%
General/nutrition	4	13%
Mental health	11	36%
Other (asthma, neonatal, otitis media)	3	10%
Smoking cessation	2	6%
Substance misuse	5	16%
**Population**		
Consumers	27	87%
Healthcare workers	4	13%
Adolescents	8	25%

**Table 4:** Multi-way cross-classification matrix: health focus, delivery mode and intervention by country

Health focus	Delivery mode	Intervention	No. of studies		
			Aust	NZ	USA
Mental health	Service provider	R U Appy (Bennet-Levy)	1		
		DM-DS(Starks)			1
	Self-administered	Sparx (Fleming; Shepherd(2))		3	
		iBobbly (Povey*; Tighe)	1.5		
	Administered with HCP support	Stay Strong (Dingwall (2); Povey*; Bird)	3.5		
		Stayin on Track (Fletcher)	1		
Harmful alcohol and illicit drug use	Self-administered	Therapeutic education system (Campbell)			1
		SBIRT (Gorman; Montag)			2
		e-SBINZ (Kypri)		1	
		Hawk 2 (Raghupath)			1
Smoking cessation	Self-administered	SmokingZine (Bowan; Taualii)			2
Cardiac	Service Provider	PREDICT-CVD (Riddell; Whittaker)		2	
	Administered with HCP support	CSIRO Cardiac rehab (Bradford)	1		
Diabetes	Self-administered	Kaya Tracker (Robertson)			1
	Administered with HCP support	Stanford IDSMW (Johnson)			1
		My Care Team (Levine)			1
General health/ nutrition	Self-administered	Gigiigoo’inaan (Dellinger)	1		
		SSB (Tonkin)			1
		Wellness mHealth Program (Verbiest)	1		
	Administered with HCP support	Diet Bytes (Ashman)		1	
Other: • Asthma • Sudden Infant Death • Otitis Media	Service provider	GASP (Ram)		1	
	Self-administered	Baby Essentials online (Cowan)		1	
	Non-interactive	Otitis Media MMS (Phillips)	1		
*Total health foci: 9*	*Total programmes for service providers: 4* *Self-administered: 12* *Non-interactive: 1* *Administered with assistance: 6*	*Total individual programmes: 23*	*Povey includes two programmes.		

### Types of WBTI used

The types of WBTI described in the source publications are presented in Tables 4 and 5. The majority of the interventions used in these studies were interactive websites (*n* = 21, of 25 interventions in the 32 studies, 84%), providing education modules and tutorials [15, 16, 19, 22, 25–27, 29, 36], screening and assessment tools [18, 21], management and monitoring tools [20, 23, 28, 34, 38], gamified avatar-based cognitive behaviour therapy (CBT) [31, 32] and decision support tools [24, 30]. Seven sources reported the use of mobile “apps” [17, 33, 39, 41–43, 45], two sources incorporated the use of text or multimedia messaging service [35, 40] and two sources studied an intervention that used a mixture of intervention tools [37, 44].

**Table 5:** Summary of research findings from included publications

First author (year) Country Funding	Study population and context	Aim/method and health condition	Intervention and delivery type	Measured impacts/outcomes (results)	Explanations provided for uptake and effects	Authors’ conclusions/recommendations
Ashman (2016) [35] Australia Funding: NHMRC University of Newcastle	27 women, 8 Indigenous (aged >17, gestation <25 weeks), median age 28. 8 regional outpatient settings (hospital, GP clinics, community organisations), New South Wales (NSW)	**Aim**: To use smartphones to take detailed assessments of dietary intake, and provide personalised online feedback, with dietician consultation. **Method**: Quantitative evaluation study. The women collected image-based dietary records, completed 24-h food recalls and a food frequency questionnaire, as well as 3 online surveys.	12-week study of “Diet Bytes” and “SNaQ” methods. Smartphones used to record dietary intake and participants received a video with personalised advice comparing their intake to the Australian Guidelines, then phone contact with a nutritionist.	*N =* 17, 77% reported dietary changes as a result of feedback. Core food group consumption was reported separately for Indigenous women. Intake was close to recommendations for fruit and dairy but below for grains, cereals, vegetables and meat. All Indigenous women met recommendations for unsaturated spreads and oils.	Based on the personalised feedback, some ate more foods from core food groups, others consumed less sugary drinks or “junk” foods. Others changed cooking methods Less than half the participants felt they had enough information about healthy eating at the time of enrolment.	The Diet Bytes method for nutrition assessment combined with the SNaQ tool for analysing nutritional content, with the provision of personally tailored feedback, may be a useful method for dietitians to assist women in optimising their food and nutrient intakes during pregnancy.
Bennett-Levy (2017) [36] Australia Funding: Australian Federal Government	26 health professionals (21 Indigenous) in regional centres in northern NSW.	**Aim**: To identify the barriers and enablers of e-mental health (e-MH) uptake amongst mainly Aboriginal and Torres Strait Islander health professionals. **Method**: Qualitative evaluation study. Trainers provided written reports and were interviewed. These data sources were analysed thematically.	A 3- or 2-day e-MH training programme entitled: “R U Appy” was followed by up to 5 consultation sessions (mean 2.4 sessions). Training focused on download and use of apps, with a focus on the app “Stay Strong” on one of the days.	Uptake of e-MH in the consultation group was moderate (22–30% of participants). Barriers to uptake were grouped into two categories: “Organisational” and “Participant perceptions”. Enablers were also grouped into two categories: “Organisational” and “Positive experience of the consultations sessions.	Features of the organisation acted as both barriers and facilitators to uptake. Where senior staff were supportive, uptake was greater. A good match between resources and work role led to excitement amongst participants about the possibility of using YouTube clips and apps for health education.	Researchers should broaden their focus and definitions of e-MH, emphasising the educational potential of resources, as well as therapeutic potential. Developing criteria for evaluating apps may promote uptake.
Bird (2017) [37] Australia Funding: Australian Federal Government	16 Aboriginal service providers in the health and community-service sectors in regional/rural areas of northern NSW.	**Aim**: 4–8-month follow-up on use of e-MH resources following an e-MH training programme and to determine what types of e-MH resources they used. **Method**: Qualitative evaluation study. 16 semi-structured interviews were transcribed and thematically analysed.	This study was a 4–8-month follow-up of “R U Appy” training, which involved a 3- or 2-day e-MH training programme then up to 6 monthly skills-based consultation sessions delivered face-to-face.	9 of the 16 service providers were using e-MH in their practice for a variety of purposes, including supporting social inclusion, self-care, education, referral, assessment, crisis response, and case management. Use tended not to include treatment of depression/anxiety.	Participants preferred e-MH resources that were easily accessed via mobile devices. Resources for treating anxiety/depression were not preferred, perhaps due to the professional backgrounds of participants, or because mental health concepts were not culturally relevant.	Workforces have characteristics that affect the uptake and use of e-MH resources. There is a need to foster production of culturally relevant resources to support SEWB and treat mental health disorders
Bowen (2012) [15] United States of America (USA) Funding: National Cancer Institute	113 American Indian (AI) youth (12-18 years) recruited during a 6-week residential summer camp for American Indian students (6^th^ to 12^th^ grade) in Rapid City, South Dakota.	**Aim**: To evaluate a smoking prevention and cessation intervention **Method**: Randomised control feasibility trial with 1-month follow-up. After baseline assessment, students were randomised to have regular access to the site or not. All participants completed a follow-up assessment 1-month post randomisation.	Self-directed educational programme (“SmokingZine”). Participants were encouraged to access it as often as they like over six weeks and had daily 1-h computer time. Modules covered education, behaviour change goals, positive values and identifying barriers to change.	52% uptake in intervention arm. The intervention did not directly affect smoking behaviour but did alter intentions to use tobacco amongst never-smokers.	Small sample, lack of statistical power. Selection bias- high grade point average and low rate of smoking at baseline. Limited access to the Web at the camp may have reduced engagement. A “group design” for future programmes where youth can use the site together.	Partial support for the potential of the tool for future research, and support for the feasibility of future research on smoking cessation programmes designed for American Indians and Alaska Natives.
Bradford (2015) [38] Australia Funding: Commonwealth Scientific and Industrial Research Organisation (CSIRO)	Non-indigenous researchers from the CSIRO consulted: staff of 1 remote Aboriginal health service; 1 urban Indigenous health institute; and cardiac/ Indigenous health specialists.	**Aim**: To adapt an established mobile phone delivered cardiac rehabilitation programme for Indigenous people. **Method:** Consultation with stakeholders and employment of Indigenous production company. Paper outlines the changes made following consultation.	Smartphone app to delivered over 6 weeks to provide cardiac rehab in the patient’s home in line with their lifestyle. Includes clinical portal, mentoring and educational material.	Modifications included flexibility in the duration of delivery, inclusion of positive measures, use of mentors, a meeting place for using the app and taking physical measures, changes to educational content words, look and feel.	Smartphone programme not yet tested but based on previous research with mainstream populations, the authors anticipate positive results. The adapted smartphone app is ready for further community consultation and trial.	Mobile delivery significantly improves primary outcomes over traditional cardiac rehabilitation care and if replicated in Indigenous populations, the programme has the potential to significantly improve life expectancy
Campbell (2015) [16] USA Funding: National Drug Abuse Treatment Clinical Trials Network; National Institute on Drug Abuse (NIDA)	*N =* 40 AI/ Alaska Native (AN), mean age 37.5; 47.5% women, recruited via two urban outpatient drug treatment programmes, Northern Plains and Pacific North-west regions.	**Aim**: To test acceptability of a web-based version of a therapeutic education system (TES) for drug treatment. **Method**: Participants completed baseline assessments then 1 week after the intervention phase, follow-up assessments plus qualitative interview.	Self-directed and comprised 32 interactive, multimedia modules based on the Community Reinforcement Approach (CRA). Delivered over 8 weeks on computers in the treatment settings.	TES was acceptable across seven quantitative indices. Qualitative findings indicated (1) content was relevant and (2) acceptability would be enhanced by better AI/AN representation across several content domains, and removal of content that is counter to AI/AN culture.	Acceptability of modules varied according to personal characteristics or experiences of participants, including perceived discrimination and “ethnic experience”. Participants rated those modules with STI/HIV information most highly.	Evidence-based, culturally informed, web interventions may address barriers to treatment in AI/AN communities. Adaptations can be made without losing fidelity. Research should incorporate cultural acceptability and a wider range of implementation issues.
Cowan (2013) [26] New Zealand (NZ) Funding: NZ Ministry of Health	*N =* 2683 completed “sessions” of use of internet link and confidence rating in. *N =* 207 Maori people completed the program.	**Aim**: to describe use, impact and reach of an online education tool for preventing sudden infant death. **Method**: online tool promoted widely, and basic usage data collected online.	Baby Essentials Online was a self-directed education, 1~15-min session of 24 slides, followed by assessment of “increased confidence” (IC)	Of Maori participants, 53 rated their IC as “low” and 154 rated their IC as “high.”	The greater IC in Maori with no greater time per slide may reflect lower starting knowledge and confidence	Help reducing SUDI in the Maori population, the online tool extended education opportunities beyond the traditional face-to-face delivery mode and is cost-effective
Dellinger (2018) [17] USA Funding: National Institute for Environmental Health Sciences; Sault Ste. Marie Tribe of Chippewa Indians	*N =* 24 (13 women) Anishinaabe (Native American), aged 25-55+, Great Lakes region.	**Aim**: To describe the development and acceptability of an app “Gigiigoo’inaan”, which aims to improve nutrition via personalised, culturally tailored advice on fish contaminants. **Method**: Mixed methods-qualitative and quantitative (survey) feedback obtained during focus groups.	Gigiigoo’inaan [Our Fish] is an app delivered on mobile phone and/or internet that provides personalised risk and benefit information on fish species based on user input information.	61% said they would consume more fish if they had regular access to the app; 75% agreed the app was useful, culturally appropriate and helped them identify fish to eat. However, some reported confusion about encouragement to eat fish combined with warnings re contaminant levels.	Negative emotions relating to app compounded by: (1) historical distrust; (2) the potential for emotional harm (disproportionate to the actual risk) from learning of above average exposure; (3) concerns that the data may misused to stigmatise Anishinaabe culture, and (4) an attitude of communal privacy.	Testing of the pilot software demonstrates the value of designing culturally adapted risk communication with vulnerable populations. The app may help to regain community interest and faith in natural resources. The findings support the assumption that the community seeks to promote the stewardship of natural resources.
Dingwall (2015a) [39] Australia Funding: Australian Federal Government	*N =* 138 (70% women, 35% Indigenous), aged 19-74 (M = 40.41, SD 12.86) service providers working with Indigenous people in the Northern Territory	**Aim**: To evaluate awareness, knowledge and confidence in e-mental health and the AIMhi Stay Strong App **Method**: Pre-post questionnaires on confidence and use of e-mental health tools with Indigenous clients.	Face-to-face training programme “Yarning about Indigenous mental health using the AIMhi Stay Strong App”. Duration not provided.	Significant improvements across all measures of skill and knowledge except for confidence in using computers	Limited awareness of e-mental health tools prior to training. The increase in confidence and knowledge post-training is promising but it is not known whether this will translate into use.	E-mental health tools have potential to improve access to culturally appropriate mental health care for Indigenous Peoples with minimal training but more research required into uptake and use.
Dingwall (2015b) [43] Australia Funding: Australian Federal Government	15 service providers from rural and remote health services working with Indigenous people in the Northern Territory,	**Aim**: To assess acceptability, feasibility, and appropriateness of a new e-MH resource for service providers. **Method**: semi-structured interviews about barriers, enablers, acceptability and feasibility of use	Clinician-assisted, Interactive app (AIMhi Stay Strong). A brief intervention focusing on worries and strengths, and enabling personal and behavioural goal- setting	Positive feedback on all aspects of the app. Thematic analysis revealed support for the acceptability, feasibility, and appropriateness of the resource amongst service providers	Simple language and visual appeal were identified as strengths. Participants indicated that the app would be particularly useful for client engagement, and that it enabled a client-centred approach. Barriers to use include access to power and internet.	e-MH interventions are likely to make an important contribution to overcoming the burden of poor service access for remote Indigenous clients including new delivery ways for health in remote regions
Fleming (2012) [27] NZ Funding: NZ Ministry of Health; NZ Tertiary Education Commission	*N =* 32 adolescents aged 13-16: 34% Maori; 38% Pacifica; 56% male completed SPARX during school class time.	**Aim**: To investigate the efficacy of the SPARX programme for symptoms of depression **Method:** Randomised wait-list control trial. Immediate vs delayed treatment (5 weeks). 10-week follow-up	SPARX comprises 7 30-min self-administered modules. 1–2 completed / week. CBT-based content including relaxation, problem solving, activity scheduling, challenging negative thinking and social skills.	Reductions in depression from baseline to week 5 compared to control, changes sustained 10-week follow-up. No significant changes in anxiety, locus on control or quality of life.	Good completion rates attributed to graphic interface specifically designed for young people and delivery during class time. Good uptake occurred where the programme was opt-out (i.e. delivered as part of school curriculum) rather than volunteer.	Delivery online and in school helped overcome embarrassment- a known barrier to help-seeking. SPARX has promise as an intervention for young people who may be reluctant to engage in traditional health services.
Fletcher (2017) [44] Australia Funding: Young and well Cooperative Research Centre; University of Newcastle	*N =* 20 Aboriginal fathers aged 18-25 recruited through ACCHS and community networks in urban, regional and rural locations.	**Aim:** To test the acceptability and feasibility a website with tailored support to young fathers and to adapt and test a mobile phone-based text message and mood-tracker program. **Methods**: Participatory qualitative methods including: “yarn-up” discussions, filming fathers’ stories, and SMS messaging. Participants and community gave feedback on all aspects.	Stayin on Track is a website with information, films and SMS messaging for young fathers. SMS was used to monitor mood and send encouraging messages. Participants were supported by senior mentors. The website was promoted via community networks and ACCHS staff.	Links sent by SMS on parent routines and “baby talk”. Information on crying, post-natal depression and bonding for dads were not highly accessed. Most participants reported positive mood (91.5%). Community feedback was positive.	Key to the success of the programme was the close research partnership with the communities involved, and the involvement of the fathers in developing the website content, and the involvement of mentors. Online delivery can help to overcome barriers to access to culturally appropriate resources.	Providing tailored online resources to Aboriginal fathers is feasible and acceptable. Through their involvement in the project, the young fathers saw themselves as mentors who could support other young men, thus enhancing project sustainability. Authors recommend refining the mentoring model and conducting further evaluation.
Gorman (2013) [18] USA Funding: source not provided	*N =* 21: 15 AI/AN women of child-bearing age representing 9 tribes, and 6 key informants in California.	**Aim**: To modify and evaluate a mainstream web-based behavioural intervention (SBIRT) on prenatal alcohol use for AI/AN women. **Method**: semi-structured focus groups and interviews. Data were transcribed; cross-case inductive analysis was used to identify themes.	Self-administered Web-based programme for screening and prevention of prenatal alcohol use, with or without personalised feedback.	5 themes: Make the programme relatable; stress confidentiality; incorporate family/ community focus; tailor content to community; and include information on health effects for children.	Effectiveness not known. Participatory development processes were essential for building relationships and trust in context where there is low trust of research.	This programme has the potential to provide a culturally appropriate, cost-effective approach to assess and prevent prenatal alcohol use.
Jernigan (2011) USA [19] Funding: source not provided	*N =* 54: 27 Native American (AI/AN) representing 18 tribes in urban and reservation settings, and 27 non-Native) participants. All had diabetes, 86.5% female. Participants recruited online.	**Aim**: to examine the feasibility and cultural appropriateness of the Stanford Internet Diabetes Self-Management Workshop (IDSMW) with AI/AN population. **Method**: Mixed methods process evaluation.	A 6-week peer-led internet-based workshop covering nutrition, complications, medications and managing emotions. Participants log in three times/week for 2 h including reading online content.	23 AI/AN participants participated regularly, 4 sporadically. Feedback indicated workshop was culturally acceptable because of the participation of AI/AN people, and that all AI/AN discussion groups were preferred.	The intervention is adaptable due to a peer-led mechanism of delivery. It was considered culturally appropriate with limited adaptation. Participatory approaches to recruitment facilitated implementation.	It is feasible to implement an Internet-delivered disease self-management workshop within a diverse AI/AN population. Several participants volunteered to be peers in future online workshops.
Kypri (2013) NZ [28] Funding: NZ Alcohol Advisory Council	*N =* 2355 Maori students aged 17-24 at seven of NZ’s 8 universities were screened for harmful alcohol use (AUDIT-C). Those screening positive (*n =* 1789) recruited to the research trial. *N =* 850 control, *N =* 939 intervention.	**Aim**: to test the effectiveness of a web-based alcohol screening and brief intervention (e-SBINZ) for hazardous drinking **Method**: Parallel, double-blind, multi-site, randomised controlled trial. Follow-up questionnaire 5 months post-randomisation.	Web-based alcohol assessment and personalised feedback on health risks, other risks, expenditure and comparative data, as well as tips to reduce harm. The intervention took <10 min post screening.	Relative to controls, participants receiving intervention drank less often, less per drinking occasion, less overall and had fewer academic problems. These differences were statistically significant.	It is possible to reach large numbers of Maori people with hazardous drinking via the internet. E-SBINZ is extremely low cost. Personalised feedback avoided framing Maori student drinking in terms of deficit.	e-SBINZ reduced hazardous and harmful drinking amongst non-help seeking Maori students and has the potential to lead to ongoing public health benefit in the long term, especially with annual implementation in all new Zealand universities. Further generalisability not clear.
Levine (2009) [20] USA: Funding: US Army. COI: 2 authors own stock (<5%) in the company that has licensed MyCareTeam technology.	*N =* 109 AN (>18 years) with type 1 & 2 diabetes mellitus recruited via Indian Health Centers in Alabama, Idaho, and Arizona. Gender/age not reported.	**Aim**: To test whether interaction with a web-based diabetes management app: (MyCareTeam®) increased monitoring of blood glucose (BG) levels and health care provider (HCP) interaction. **Method**: non-randomised prospective feasibility study.	The app that provided feedback on blood glucose levels, culturally adapted information, and facilitated timely interaction between patients and HCPs through text messaging.	Use of the app varied, with 46/109 using it 2/month. The more participants used the app, the more they tested their BG and interacted with their HCP. The messages from HCPs seemed to help motivate use of the app.	One of the key mechanisms by which the app worked was increasing the sense of closeness between the patient and HCP, increasing trust and accountability. The authors suggest that apps without this personal element may not be as effective.	Use of the app encouraged HCP-patient interaction and patient-centred communication, which in turn increased BG monitoring. Authors suggest further research on the relationship between messaging and clinical health benefits.
Montag (2015) [21] USA Funding: National Institute of General Medical Sciences	*N =* 263 AI/AN women of child-bearing age in Southern California, recruited via health clinics. *N =* 121 intervention *N =* 142 control (TAU)	**Aim:** to assess the effectiveness of SBIRT at reducing risky drinking and risk of alcohol-exposed pregnancies (AEP). **Method**: Randomised trial with follow-up questionnaires at 1, 3 and 6 months.	SBIRT was an adapted from eCHECKUP TO GO, a brief (20 min) intervention comprising: web-based survey with personalised feedback including analysis of risk, and helpful advice that could be printed out confidentially.	No difference between intervention and control groups. Risky drinking decreased in both groups: drinks/ week, (*p* < 0.001); frequency binge episodes/2 weeks, (*p* = 0.017) and risk of AEP (*p* < 0.001) at 6 months post intervention.	Baseline factors associated with decreased alcohol consumption at follow-up included the thinking other women group drink more, more binge episodes in the past 2 weeks, needing treatment for depression.	Null finding suggests that assessment alone, without intervention, may be enough to decrease risky drinking and vulnerability to AEP. Contraceptive could be added to future interventions to reduce vulnerability to AEP.
Phillips (2014) [40] Australia Funding: Australian Government Department of health and Ageing Hearing Loss Prevention Program.	*N =* 53 (30 intervention, 23 control) caregivers of Aboriginal children living in remote community households in NT, with access to a mobile phone in the household.	**Aim**: To test whether WBTI for families of children with tympanic membrane perforation (TMP): (i) increased clinic attendance, (ii) improved ear health and (iii) provided a culturally appropriate method of health promotion. **Method**: multi-centre, parallel group, RCT.	One ear health Multimedia Media Service (MMS) in the local Indigenous language sent every 4 days, ±24 h window over 6 weeks. Videos were short, animations of Indigenous role models, accompanied by personalised text messages in English with a prompt to visit the clinic.	No significant difference between groups in clinic visits per child, healed perforation, middle ear discharge or perforation size. Majority were happy to receive the messages. Ten families in the intervention group reported not receiving the messages.	Culturally appropriate MMS that could be shared amongst families, the video messages may have been unclear or confusing, and simple text messages may be more effective. Uptake was impacted by events in the community unrelated to the trial.	Mobile phone-based MMS and text messaging intervention was acceptable, but it had no short-term impact on clinic attendance or ear health. A study over a longer time period may be more informative 4`.
Povey (2016) [41] Australia Funding: Northern Territory (NT) Department of Health	*N =* 9 (3 male; 18–60 years old) Aboriginal and Torres Strait Islander community members without serious mental illness in Darwin, NT.	**Aim**: To explore acceptability of two culturally responsive e-mental health apps. **Method**: 3 3-h focus groups. Transcripts were member-checked and analysed thematically.	The AIMhi Stay Strong iPad app is a clinician-assisted therapeutic goal setting tool. iBobbly is a self-help suicide prevention app for mobile device based on acceptance commitment therapy.	Findings indicated that acceptability was influenced by characteristics of the person (e.g. mental health), environment (e.g. stigma) and apps (e.g. ease of use.	Uptake and use were reportedly influenced by motivation to change; technological competence; literacy and language; internet or phone access; free download; ease of navigation; cultural relevance, voices, animations.	E-mental health tools can improve the wellbeing of Indigenous people. There was strong support for the concept of e-mental health apps and optimism for their potential. Specific adaptations may aid uptake.
Raghupathy (2012) [22] USA Funding: National Institute on Drug Abuse	Rural and urban AI/AN youth, other service providers and artists collaborated on the development process in northern California. *N =* 45 AI/AN youth aged 11-13 participated in the final review.	**Aim**: To describe the adaptation of a drug prevention intervention into a low-cost computer-based drug prevention intervention: Honouring Ancient Wisdom and Knowledge (HAWK2) **Method**: Descriptive review of development process	HAWK2 comprised 7 lessons, 25–30 min each, which could be implemented flexibly. Total exposure 3.5 h. Evaluation at the end of each lesson	In the final review with, HAWK2 received high mean ratings on likeability (4.8/5), ease of use (4.5/5), comprehension (4.6/5), and future use (5.0/5). Practitioners also gave positive feedback.	Strengths were: Recognising the influence of specific cultural and contextual variables; building on an existing evidence-based program; and Integrating community perspectives.	Computer-based interventions are a cost-effective way of engaging youth in prevention programming. Future studies of effectiveness and feasibility are needed.
Ram (2014) [29] NZ Funding: Asthma Foundation of NZ	*N =* 761 consecutive patients and 18 nurses in primary care. *N =* 44 were Maori patients, *n =* 18 Pacifica. Age ranged from 5 to 64 in the Waitemata region of Auckland.	**Aim**: To evaluate the effectiveness of the online intervention at reducing exacerbations, hospital admissions and emergency presentations, use of corticosteroids and bronchodilator reliance. **Method**: Retrospective cohort study. Patient data were compared pre-post intervention.	GASP is an online decision support tool for primary care, providing service providers with skills & knowledge to undertake a structured asthma assessment. The GASP tool is also shown to patients.	Maori and Pacifica patients showed a significant decrease in ED presentations but no differences in risk of exacerbations, use of other treatments or hospital admissions. Asian and NZ European patients showed benefit on all measured outcomes.	The difference in benefit may be attributed to the significantly greater burden of respiratory illness in Maori and Pacifica and Maori populations, including a hospital admission rate twice that of New Zealand Europeans.	GASP in primary care has the potential to translate into significant clinical improvements for but its potential for use in Maori and Pacifica populations needs to be further explored.
Riddell (2007) [30] NZ Funding: Health Research Council of NZ; National Heart Foundation	*N =* 19,164: Maori = 1450 (7%). Mean age: 53.2, 46% female. Participants attended “ProCare” primary health care providers in Auckland.	**Aim**: To describe the cardiovascular disease (CVD) risk factor status and risk management of Maori vs non-Maori using PREDICT-CVD **Method**: Patients opportunistically assessed in routine primary care practice.	PREDICT-CVD is a web-based clinical decision support programme for CVD risk assessment and management. It has been shown to increase CVD risk assessment rates in primary care.	Maori were assessed 3 years younger than non-Maori. Maori with CVD received more anti-coagulants, BP-lowering and lipid-lowering medications. Maori with Ischemic heart disease were half as likely to have a revascularisation procedure.	An electronic decision support programme generated CVD risk burden and risk management data for Maori and non-Maori populations in routine clinical practice in real-time.	When Maori specific equations replace those based on a white, middle-class American population, the PREDICT-CVD will provide a world-class data system that can identify gaps in care for Maori patients and enable action on them.
Robertson (2007) [23] USA Funding: South Dakota State University Foundation	*N =* 52 Lakota Sioux AN with type 2 diabetes individuals (33 intervention group, 19 controls), living on Northern Plains Indian Reservation, Sioux Falls, South Dakota.	**Aim**: To develop and test a culturally appropriate web-based interactive programme (Keya Tracker) for management of type 2 diabetes. **Method**: Randomised control trial. Pre-post data collected on HbA1c, exercise, diet, cultural activities, and social activities.	Kaya Tracker was an interactive website developed with input from tribal Elders. Content covered nutrition, physical activity, social and cultural activities. Participants logged-in 3 times per week for 24 weeks.	HbA1c control improved in the intervention group relative to controls (p = .025), suggesting improved disease control and programme effectiveness. Four participants did not complete the intervention.	Effectiveness may be due to the flexibility of the online delivery. Also, the website was designed for its audience, therefore accounting for Lakota Sioux understandings of health.	Use of a culturally appropriate Web-based interactive programme may be a viable tool to assess with diabetes-related lifestyle change. A larger study is warranted.
Shepherd (2015) [31] NZ Funding: NZ Ministry of Health; Rotary Club of Downtown Auckland; University of Auckland. COI: 2 authors have financial interest in SPARX.	*N =* 26 Māori people, taitamariki (adolescents); taitamariki mothers (aged 16–18); and whanau (family) in Auckland.	**Aim**: To describe experiences of a prototype computerised therapy programme for treating mild to moderate depression. **Method**: Mixed method Kaupapa Maori research. 7 focus groups followed by a survey. General Inductive/thematic analysis was used to generate themes.	Smart, Positive, Active, Realistic, X-factor thoughts (SPARX) provides free, computerised (cCBT); online computer programme using avatars. Players are led through 7 fantasy “realms” each lasting 30–40 min. 1–2 levels completed over 3–7 weeks.	Good face validity; cultural relevance for Maori; Whanau are important for young people’s wellbeing. Ideas for improvement related to use of clinical and Maori language, reducing text, and using audio.	Positive evaluation and acceptance were aided by cultural relevance of both process and content. Culturally adapted mental health interventions are thought to be much more effective.	Participants supported the contemporary Maori design of the program. SPARX was the first programme of its kind and may be used as a model for other cCBT interventions.
Shepherd (2018) [32] NZ Funding: NZ Ministry of Health; Rotary Club of Downtown Auckland; University of Auckland; Te Rau Matatini	*N =* 6 Māori taitamariki (adolescents) aged 14-16 (mean 14.6), in two schools in the wider Auckland area. Participants had mild-moderate depression and low risk for self-harm.	**Aim**: To explore adolescents’ opinions about a programme for treating mild/moderate depression in young people **Method**: Exploratory qualitative study using semi-structured interviews. Thematic analysis.	The SPARX programme is an online, gamified cCBT programme using avatars for treating mild – moderate depression.	Themes indicated that: (1) the programme was helpful because it taught CBT skills; (2) It was engaging due to Maori designs; (3) The characters provided helpful advice; (4) It was both enjoyable and challenging; 5) Writing thoughts and feelings was helpful.	Māori designs appeared beneficial, as this seemed to enhance cultural identity. SPARX was like a computer game that could help with depression. A breathing relaxation exercise was valued	Māori designs were appropriate and useful. The ability to customise the characters with Māori enhanced cultural identity. A much larger study should be conducted to explore the efficacy of SPARX.
Starks (2015) [24] USA Funding: Patient-Centred Outcomes Research Institute	Multiple groups of stakeholders consulted within South Central Alaska Native Foundation had input into tool development. *N =* 20 patients and 7 service providers participated in piloting the tool.	**Aim**: To report on the multi-year stakeholder engagement process for the development of the patient-centred “Depression Management – Decision Support Tool (DM-DST)”. **Method**: Qualitative analysis of multiple data sources including interviews with patients and providers, meeting notes, consultations and pilot testing.	Electronic, patient-centred, depression management decision support tool (DM-DST) with two components: an interactive tool to facilitate discussions between patients and providers and a website with detailed information for patients.	Stakeholder engagement resulted in substantial modification of the original tool, including breaking it into two parts, incorporating AN imagery and cultural concepts including faith, family and cultural expressions of depression and solutions. There was a focus on reducing stigma.	Multi-stakeholder engaged research allowed the researchers to understand the diverse values and needs of end-users. The tool was considered interesting, useful, and thought-provoking with the potential to foster conversations with primary care providers.	The process employed as relevance to other primary care systems seeking to improve and individualise treatments. The tool enhances patient-centred decision making. Future research will test its effectiveness is an RCT.
Taualii (2010) [25] USA Funding: Spirit of Eagles Special Populations Network (NIH)	*N =* 25 urban American AI/AN young people, aged from 12-18 in Seattle.	**Aim**: To adapt, modify and test for useability an existing smoking prevention and cessation resource. **Methods**: Focus groups were conducted in 2 phases, first to adapt and then to test the usability of the SmokingZine website.	SmokingZine an e-health website targeting behaviour change relating to youth smoking prevention and cessation.	Participants were receptive to the use of the intervention tool and offered ideas for changes to make it more culturally relevant. In phase 2, participants found the site easy to use and relevant to smoking cessation.	There was a lot of overlap between mainstream and AI/AN youth perspectives on smoking. Including cultural distinctions in a new website was acceptable and valued, although computer access not ubiquitous.	These findings provide justification for a full-scale trial of the SmokingZine website. Future research should include both urban and rural AI/AN youth and consider delivery through schools.
Tighe (2017) [42] Australia Funding: Australian Government Department of Health and Ageing	*N =* 62 young men (22, 36%) and women (aged 18–35 years) in remote communities in the Kimberley region of north-Western Australia. 4 were non-Aboriginal, the remainder were Aboriginal and/or Torres Strait Islander.	**Aim**: To evaluate the effectiveness of a self-help mobile app for suicide prevention. **Method**: Randomised waitlist control trial. Measures were taken face-to-face at baseline and after the intervention for both groups. The control group had a final follow-up assessment at 12 weeks.	iBobbly was a mobile app that targets suicidal ideation, depression, psychological distress and impulsivity using Acceptance Commitment Therapy approaches. Three content modules and three self-assessments, completed over 6 weeks.	Data were available for 40 participants. Significant pre/post changes on suicide ideation in the iBobbly arm (*p* = 0.0195), but not when compared with waitlist arm. iBobbly group showed reductions in depression and distress scores compared with waitlist. Waitlist improved after 6 weeks of app use.	Uptake was aided by the collaborative development process. Acceptance was indicated by the promotion of the app by the target community. Technical/ connectivity failure prevented some from providing final data.	The app, using acceptance-based therapy reduced distress and depression but did not show significant reductions on suicide ideation or impulsivity. Study highlighted the importance of co-design.
Tonkin (2017) [45] Australia Funding: NHMRC; National Heart Foundation; NT Government Department of Health	*N =* 36 smartphone users (aged 18–35 years) in two remote Indigenous communities in NT. There were 10 participants per community in each phase of research.	**Aim**: To develop and test a prototype app to improve nutritional intake relating to sugar-sweetened beverages (SSB). **Method:** Formative phase included simulated grocery selection activity, semi-structured interview, and survey. End-user testing phase involved a “think aloud” test and interview on user satisfaction.	Self-administered Smartphone app including assessment and feedback on SSB intake, behavioural challenges, interactive exercises and games.	Drivers of food choice/behaviour included taste, family, health, price and convenience. Mixed methods data on usability indicated that participants found the app useful & were confident using it, with some suggested modifications.	Complex set-up and log-in inhibit use. Dissemination of apps should be contextually embedded with many avenues available. Learnings about the social dynamics of remote communities in this study may have relevance to other disadvantaged communities.	Recommendations included: formative research needs to be prioritised in project plans; use mixed methods; patterns of technology use may be different in different locations; include non-written communication; local language; engaging graphics.
Verbiest (2018) [33] NZ Funding: Healthier Lives *He Oranga* Hauora National Science Challenge	Partnership between Maori, Pasifika and European academics, Maori health providers and community members in Wellington and Auckland regions.	**Aim**: (a) to provide overview of co-design methods and processes; (b) to describe how co-design was used to select behavioural determinants and change techniques. **Method**: 6-step participatory co-design process conducted over 11 months. Focus groups, photographs, notes and observations were thematically analysed.	Self-administered mHealth tool (smartphone app) for prevention of non-communicable disease, incorporating contemporary Maori and Pasifika theoretical frameworks of health and health promotion.	Domains prioritised: (a) physical activity, (b) family, and (c) healthy eating (including fruit and vegetable gardening; Table 1). Māori community partners identified additional ethnic-specific themes relevant for overall Māori health and wellbeing.	By using ethnic-specific models of health for interpreting the co-design data, the selected behavioural barriers, enablers, and change techniques align with the cultural needs and wants of the user.	Authors suggest future tailored, lifestyle support (mHealth) interventions for Indigenous and other priority groups should be co-designed and look beyond Western approaches to ensure they are evidence-based and culturally relevant.
Whittaker (2006) [34] NZ Funding: Waitemata District Health Board; Future Forum; National Heart Foundation	80 General Practitioners (GPs) providing care to Maori and non-Maori people in New Zealand. *N =* 474 (28.2%) Maori patients pre- and *n =* 484 (25.7%) post- intervention.	**Aim**: To determine if an electronic assessment and management tool for CVD could increase risk assessment but not inequalities. **Method**: Retrospective audit of GPs using the tool’s electronic medical records (EMRs)	PREDICT-CVD is a web-based decision support tool to facilitate risk assessment for GPs to use in the management of CVD in primary care.	Maori participants were significantly different from non-Maori on all measured parameters. Maori were younger, had more diabetes, lower SES and higher rate of smoking. Rate of risk assessment increased in both groups.	Rates of documented risk assessment were low overall. 7.2% of audited EMRs had no ethnicity stated and coded as non-Maori, which may have resulted in under- counting of Maori.	The implementation of the tool should not occur without an implementation program, and other changes to increase responsiveness to the needs of those at risk of CVD, such as taking risk assessment into community settings.

Most interventions were self-directed (*n* = 19, 61.3%), requiring the user to access the WBTI program, often according to a pre-defined schedule, without support from an outside agency or healthcare worker [16, 18, 19, 21–26, 29–34, 37, 38, 41, 42, 45]. The remainder of the interventions were either supported by a healthcare worker or other personnel [17, 20, 27, 28, 35, 36, 39, 43, 44], were supported initially but then relied on users to be self-directed thereafter [15] or were passive [40], such as the receipt of intermittent text messages that contained health messages or graphics intended to prompt a behavioural response.

### Uptake and effects of WBTI

The bulk of the sources reported improved health outcomes for Indigenous people [15, 16, 18–20, 22, 24, 28, 31, 33–38, 41]. For the three studies that reported uptake, voluntary uptake of WBTI was between 30 and 56% [15, 27, 36, 37]. The remainder of the sources had the WBTI as a prescribed component of the reported intervention or evaluation study so rate of uptake is not relevant; however, contextual factors identified by study authors as influences on uptake, use and acceptability are discussed below.

None of the WBTI approaches had a negative impact on participants. However, some WBTI were more successful than others. Two randomised clinical trials reported statistically significant differences in quantitative measures of depression amongst Indigenous adolescents (USA and NZ) who used WBTI “apps” compared to those who did not [27, 42], while a qualitative study found substantial improvements in mood amongst Maori adolescents who used a gamified app [32]. An interventional study using WBTI with Native Americans for diabetes control showed a statistically significant improvement in glycated haemoglobin levels [23], and an educational tool for preventing sudden infant death in New Zealand significantly increased the confidence of Maori people to discuss infant sleep safety with others compared with non-Indigenous people [26].

Two sources reported no impact of the intervention. In the USA, risky drinking behaviour by women living in California decreased regardless of whether they received the WBTI or “usual care” [21]. In Australia, while health messages sent via text message did not impact on clinic attendance for children with otitis media [40], the content delivered in local Indigenous languages was found to be culturally appropriate and recipients were happy to receive the information.

### Explanations for uptake

While some sources provided no explicit explanations for uptake success or failure [18, 30, 35], most did. Sources suggested a variety of factors that could improve WBTI uptake; however, the most important was ensuring that WBTI were designed for the audience [23, 44], culturally relevant and appropriate [40, 41, 43], by having culturally relevant graphics, voices and animations [40], and showing traditional practices, culture and Indigenous peoples [16, 44]. It was also important that content was matched to participants’ values and experiences. For example, Campbell et al. [16] reported that ratings of some modules varied according to experiences of discrimination or mainstream comfort. Cultural appropriateness of the WBTI was explicitly discussed and/or evaluated in all but four sources [26, 30, 34, 35]. Where authors discussed cultural appropriateness, it was linked to better outcomes in terms of acceptability, uptake and impact, whether or not this was formally evaluated. Seven sources reported recommendations from their study populations on features to improve so as to ensure the WBTI contain a greater volume of culturally appropriate content [16, 30, 33, 34, 40, 44].

In addition, sources reported that users wanted WBTI to have an innovative and visually appealing format [43] and be useful [24, 43], interesting [24] and thought-provoking [24]. These interventions fostered conversations [24] and resulted in improved patient-practitioner relationship which led to better health monitoring by patients [20, 29]. However, one source reported that videos sent via multimedia messaging services may be unclear or confusing and suggested that simple text messages may be more effective [40]. Users like the flexible delivery that WBTI allow [23, 38] and want WBTI to be easy to use [41, 43] and customisable [38]. However, users also preferred to be able to download the “app” or software for free [41]. Limited access to internet or a phone were found to be prohibitive factors by users [15, 41], with mental health workers reporting significant organisational and personal barriers to accessing mental health web-based apps [36]. Systemic or policy contexts that influenced uptake and use included the following: organisational support for the WBTI [36], access to the internet or other technology due to cost or the availability of infrastructure [15, 23, 39–41], underlying burden of disease [29] and funding to reduce the cost to end-users [31, 41].

### Recommendations and conclusions from the sources

Culturally appropriate, evidence-based WBTI have the potential to improve mental health [27, 32, 42], address substance abuse treatment barriers [16], improve self-efficacy and self-management in healthcare [23, 26] and reduce inequalities in access to healthcare services, for Indigenous communities. WBTI are a cost-effective method of delivering information and engaging target populations, such as youth [22, 28] and pregnant women [18], to reduce hazardous and harmful alcohol intake. In fact, one source reported that using a WBTI to self-assess risky drinking behaviour may be enough to influence behavioural change, without implementation of an intervention [21].

Thus far, evaluations of smoking cessation WBTI for Indigenous populations have been limited to America [15, 25]. These evaluations concluded that additional long-term, rigorous research is needed to assess WBTI approaches to keep American Indian and Alaskan Native youth from becoming regular smokers [15] and that future research needs to include both urban and rural Indigenous youth [25], highlighting that to date no online tobacco programmes have been designed specifically for these populations [15].

Sources that evaluated the use of mental health WBTI for Indigenous populations concluded that these interventions are likely to be important in overcoming poor access to services for remote communities [39, 43], or for youth that might be reluctant to engage in traditional health services [27]. Further, developing WBTI in partnership with Indigenous communities ensures culturally appropriate interventions that are accepted and promoted by the communities [24, 31–33, 42, 44], and lead to improved wellbeing of Indigenous people [41]. In addition, improved access to culturally appropriate WBTI tools, and training in how to use these tools, allows mental health workers to better support their Indigenous clients [36, 37, 39].

Cardiovascular disease risk data for Maori people can be successfully generated in real time through the use of an electronic decision support tool [30]. In addition, cardiac rehabilitation care delivered through WBTI platform has the potential to significantly improve outcomes for Indigenous populations [38]. However, other researchers suggest that the use of WBTI for cardiac care should not occur in isolation, instead emphasising that WBTI should be complemented by a comprehensive care program [34].

Implementing culturally appropriate WBTI for the self-management of diabetes has been shown to be feasible [19]. The use of such platforms has been shown to improve diabetes control in Indigenous populations [23], possibly because these interventions may increase the frequency patients monitor their blood glucose levels [20].

The remainder of sources identified for inclusion in this review also recommended WBTI as positively supporting Indigenous communities. WBTI were shown to have the potential to be a useful tool for dieticians working to optimise the food and nutrient intakes of pregnant women [35] and may reduce sudden unexpected infant deaths by increasing education beyond traditional face-to-face delivery methods [26]. Research demonstrated the importance of designing culturally appropriate WBTI to promote health through understanding and use of natural resources [17], to increase ear health knowledge [40] and to improve asthma education [29].

## Discussion

The purpose of this review was to identify and describe the available international scientific evidence on WBTI used by Indigenous peoples in Australia, New Zealand, Canada and USA for managing and treating health conditions. As mobile devices, including smart phones, become ubiquitous in the general population, web-based and other electronic interventions are increasing in number and scope. This is indicated by an increase in studies on the topic year on year, and of the many protocols that were excluded from this scoping review, but which clearly indicate a growing field with many studies planned or underway. The results indicate that while WBTI are most commonly designed to manage mental health and substance use issues, they are increasingly being incorporated in the range of treatment and support options for a variety of health conditions and are being used by those with the health condition or by service providers.

The popularity of WBTI stems in part from their potential reach, which extends to anyone in any geographical location or social context where they have access to the internet and a device capable of running the program. While increases in internet access amongst Indigenous populations in Australia, New Zealand, Canada and USA have been reported, in each of these countries, a “digital divide” across ethnic and geographical lines also exists, with Indigenous households and individuals having less access to the internet overall, and internet access being lowest in those geographical areas with the highest proportion of Indigenous residents [46]. The lack of any studies from Canada meeting the inclusion criteria was surprising, however there is evidence that other forms of ICT, such as telehealth, are in use in Canada, and that First Nations communities are engaging with and developing digital health technologies in line with Indigenous models of health and wellbeing [47].

The available data indicates that young people tend to have greater internet access and use [48] so it is perhaps not surprising that adolescents or young people were the focus of a large proportion of studies (8/34). Indigenous populations in Australia, USA, Canada and New Zealand are younger than the general populations in each country [46, 49–51], so interventions designed to improve the wellbeing of young people are particularly relevant. However, there were several studies of WBTI focusing on chronic diseases such as diabetes and cardiovascular disease that are relevant to older people.

Perhaps the most common and clear lesson articulated by study authors was the importance of developing programmes in collaboration with the target communities. In this sense, WBTI are no different to other interventions, with issues of governance and ownership being central not only to the ethical delivery of programmes but also to their acceptability, feasibility and effectiveness. Several authors cited “co-design” or collaborative design as strengths of their projects. Having the target community involved in all aspects of intervention and study design is in line with ethical guidelines for research with Indigenous peoples internationally [52–55].

Meta-analysis of effectiveness was beyond the remit of this scoping review, and while such analysis is likely to be limited by the small sample sizes evident in these studies, future research could usefully examine both the effectiveness and cost-effectiveness of WBTI with Indigenous people. Cost-effectiveness is frequently highlighted as an advantage of WBTI. However, few studies include analysis of cost-effectiveness that incorporates the often-substantial costs of development that may occur over lengthy time periods. This scoping review was limited in the degree to which it could examine barriers to accessing WBTI resulting from cultural and linguistic diversity, low health literacy, limited digital capabilities and infrastructural and resource limitations for individuals and communities in different geographic locations. This review is also based on a definition of health that is less holistic and relational than Indigenous models of health and wellbeing tend to be [1]. Future research could focus more explicitly on a broader range of social health factors, such as language use and reclamation, as these are likely to have health benefit from an Indigenous perspective [56]. Broadening the review to include digital sources, such as app stores and social media, could also provide a more comprehensive account of all indigenous-focused WBTI and tools, although formal evaluations of such interventions, which are the focus of this review, are unlikely to be sourced this way.

As mobile digital devices become cheaper and more widespread, and internet technology improves in speed and geographic coverage, it seems safe to assume that WBTI will also become more widespread, and of interest to health services and commercial entities who may wish to exploit potential markets. This is true for Indigenous peoples as it is for others, although the smaller population size may limit commercial interest in Indigenous-specific WBTI and explain the propensity of funding from government rather than commercial sources. As the field grows, ensuring that technologies accessed in Indigenous communities are high quality, evidence-based, culturally appropriate, inclusive and accessible will require that we continue to examine and re-examine the evidence as it emerges and that Aboriginal communities continue to lead the development of technologies that best meet their needs.

## Supplementary information

**Additional file 1.** Preferred Reporting Items for Systematic reviews and Meta-Analyses extension for Scoping Reviews (PRISMA-ScR) Checklist.
